# Microendoscopic calcium imaging in motor cortices of macaques during rest and movement

**DOI:** 10.1016/j.isci.2025.112767

**Published:** 2025-05-27

**Authors:** Anne-Caroline Martel, Damien Pittard, Annaelle Devergnas, Benjamin Risk, Jonathan J. Nassi, Waylin Yu, Joshua D. Downer, Thomas Wichmann, Adriana Galvan

**Affiliations:** 1 Emory National Primate Research Center, Emory University, Atlanta, GA 30329, USA; 2 Udall Center of Excellence for Parkinson’s Disease Research, Emory University, Atlanta, GA 30329, USA; 3 Department of Neurology (School of Medicine), Emory University, Atlanta, GA 30329, USA; 4 Department of Biostatistics & Bioinformatics (Rollins School of Public Health), Emory University, Atlanta, GA 30329, USA; 5 Inscopix Inc., Mountain View, CA 94040, USA; 6 Aligning Science Across Parkinson’s (ASAP) Collaborative Research Network, Chevy Chase, MD 20815, USA

**Keywords:** Behavioral neuroscience, Systems neuroscience, Techniques in neuroscience

## Abstract

The study of motor cortices in non-human primates is relevant to our understanding of human motor control, both in healthy conditions and in movement disorders. Calcium imaging and miniature microscopes allow the study of multiple genetically identified neurons with excellent spatial resolution. We used this method to examine activity patterns of projection neurons in deep layers of the supplementary motor (SMA) and primary motor areas (M1) in four rhesus macaques. We implanted gradient index lenses and expressed GCaMP6f to image calcium transients while the animals were at rest or engaged in an arm-reaching task. We tracked the activity of SMA and M1 neurons across conditions, examined cell pairs for synchronous activity, and assessed whether SMA and M1 neuronal activation followed specific sequential activation patterns. We demonstrate the value of *in vivo* calcium imaging for studying patterns of activity in groups of corticofugal neurons in SMA and M1.

## Highlights

• One-photon calcium imaging in SMA/M1 during rest or arm reaches in macaque
• We assessed dynamic activity in genetically tagged deep-layer cortical cells
• Calcium transients during reaches showed direction sensitivity in subset of neurons
• In SMA/M1, some cells coactivated or showed sequential patterns during rest/reaches

## Graphical abstract

**Figure:**
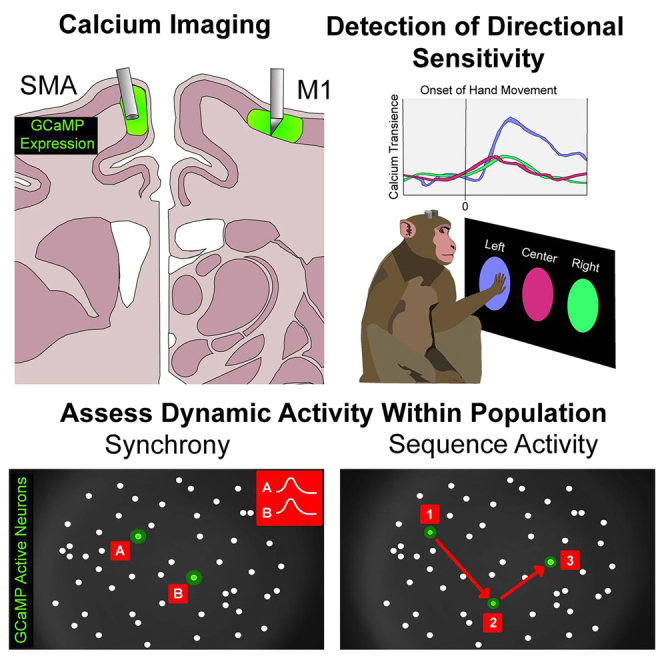

## Introduction

Cortical motor regions have undergone significant evolutionary expansion, specialization, and re-organization^1^; therefore, non-human primates (NHPs) are often used to model motor control in humans under healthy conditions and during movement disorders such as Parkinson disease. In primates, the motor cortices include the primary motor cortex (M1) and the supplementary motor area (SMA),^2^^,^^3^ which are components of larger frontal networks that also involve portions of the basal ganglia and the ventral anterior and ventral lateral thalamus.^4^^,^^5^^,^^6^^,^^7^ Neurons in M1 and SMA are involved in movement planning and execution.^2^^,^^8^

The majority of studies on cortical neuronal activity in awake NHPs have used extracellular electrophysiological recordings. Methods such as one-photon fluorescent miniature microscope (miniscope) imaging of genetically encoded calcium indicators offer an alternative approach to monitor neuronal activity and have recently been used to study calcium transients in individual cells in the premotor and visual cortices of macaques.^9^^,^^10^^,^^11^

When combined with microendoscopic gradient index (GRIN) lenses, miniscopes enable optical access to non-superficial imaging regions and allow prolonged monitoring of multiple individual neurons in the brain region of interest, providing detailed understanding of spatial relationships among cells. Calcium indicators, such as GCaMP, can be genetically directed to be expressed in selective cell populations, and the resulting calcium activities of identified neurons can be followed throughout the course of an experiment, enabling tracking of the same cells across behavioral conditions (e.g., from rest to active movement).

We used microendoscopic calcium imaging to examine the activity of neuronal ensembles in SMA and M1 in four rhesus macaques while the monkeys were at rest or performing simple arm reaches. Following expression of the genetically encoded calcium indicator GCaMP6f in projection neurons of deep cortical layers of SMA and M1, we tracked the activity of these neurons across conditions, examined cell pairs for synchronous activity or inactivity, and assessed whether neuronal activation followed specific sequential activation patterns. Our results demonstrate that calcium imaging captures dynamic activity patterns in SMA and M1 neurons and establish a basis for future research on how SMA and M1 activities change during the development of parkinsonism and other disorders of movement in NHPs.

## Results

We studied the neuronal activity in SMA and M1 in four macaques using calcium imaging. We used adeno-associated viruses (AAVs) to express GCaMP6f under control of the Thy1 promoter to target preferentially projection neurons^12^ in deep cortical layers. Surgical preparations to chronically implant microendoscopic GRIN lenses for optical access to motor cortices and to ensure a stable long-lasting cranial chamber were performed as described in Bollimunta et al. (2021). Out of four subjects, we successfully performed calcium imaging in three SMA sites (monkeys Q, U, and F) and two M1 sites (monkeys V and U).

### General description of calcium activity

Calcium imaging was done while the animals were sitting in a primate chair and making occasional spontaneous movements (“spontaneous” state) or while they were engaged in a reaching task (monkeys Q and U).

**Figure 1:**
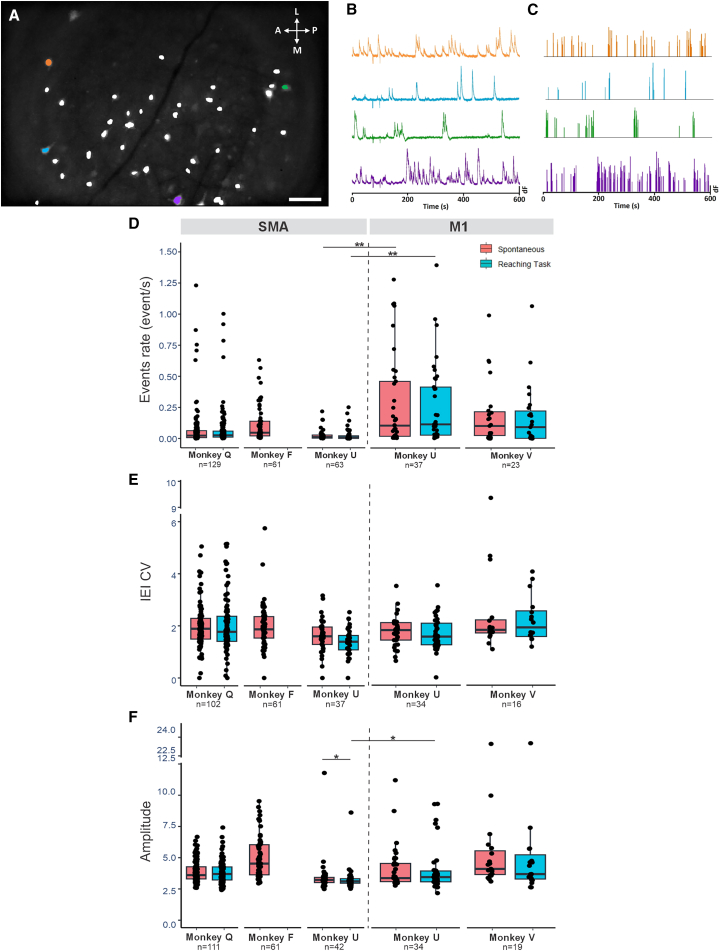
Steps of analysis and general description of cell activity (A) Maximal intensity of GCaMP6f fluorescence in the SMA during a single example session (monkey Q), overlapped on the cell map (white) extracted using CNMF-E. Orange, blue, green, and purple symbols mark the example cells shown in (B and C). Scale bars: 100 μm. (B) Raw traces of calcium transients (dF, peak-normalized) during spontaneous activity. (C) Calcium events deconvolved from the calcium traces in (B). (D–F) Boxplots summarizing the event rate (events/s), inter-event interval coefficient of variation (IEI CV), and amplitude of events detected across all sessions in the SMA (left) and M1 (right) in the spontaneous condition (red) and during the arm reaching task (blue). The horizontal bar in each box indicates the median; each circle indicates the median value for a cell. ∗*p* < 0.05, ∗∗*p* < 0.001. Wilcoxon signed rank tests corrected for multiple comparisons using the false discovery rate (FDR). See also Tables S1 and S2.

Individual cells and their calcium dynamics were identified using the constrained non-negative matrix factorization for microendoscopic data (CNMFe) algorithm and validated, based on their activity pattern and morphology (Figures 1A–1C; see Table S1 for parameters used). The number of identified GCaMP6f-expressing cells varied across imaging sites and sessions. In the SMA, we identified 129, 61, and 63 cells in monkeys Q, F, and U, respectively. In M1, we identified 37 and 23 cells in monkeys U and V, respectively (Table S2). We could track a small portion of cells (18%) across up to three recording sessions.

Calcium events were extracted from the raw calcium traces using the OASIS deconvolution method (see STAR Methods). A comparison of calcium event kinetics revealed region- and task-dependent responses in SMA and M1 neurons. The calcium event rates and the coefficient of variation of the inter-event intervals (IEI CV) did not differ between the spontaneous and reaching task conditions in either cortical region (Figures 1D and 1E). In the SMA, the amplitude of the events was significantly higher in the spontaneous state, compared to the reaching task recordings in monkey U (*p* = 0.032; Wilcoxon signed rank test, corrected for multiple comparisons). In monkey U, we found that the event rate was higher in M1 than in SMA, both during the spontaneous and reaching task conditions (*p* < 0.001 for both, Wilcoxon rank-sum test). Similarly, the amplitude of the events was higher for the task in M1, compared to SMA (*p* = 0.03, Wilcoxon rank-sum test, Figures 1D–1F).

### Calcium traces in SMA and M1 show changes in activity in relation to an arm-reaching task

We measured the calcium activity of SMA and M1 neurons in relation to behavioral events in an arm-reaching task. Monkey Q performed a simple one-target task in which a circle was randomly presented at the right, left, or center of a touchscreen. Touching the target within 3 s resulted in juice delivery. Monkey U was trained in a task in which a center target appeared, requiring the monkey to hold it for 1 s before a second target appeared on the left or the right of the screen. The monkey had to release the holding target within 1 s to receive the juice reward. We first aligned the calcium transients to the appearance of the rewarded target, as an approximation of movement onset.

**Figure 2:**
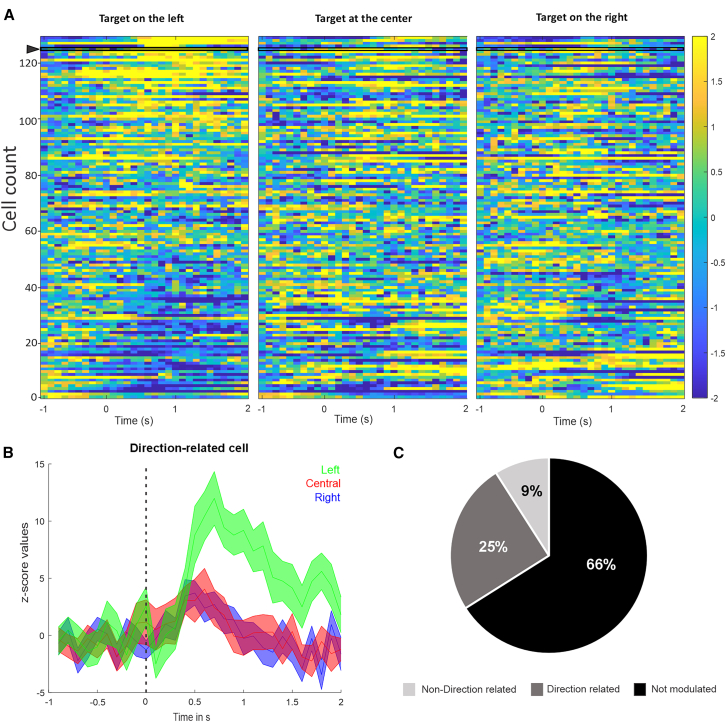
Calcium activity changes in SMA neurons related to the arm reaching task (A) Heat maps of the *Z*-scored raw calcium traces for each cell in the population, aligned on rewarded target onset on the right, center, or left of the screen during the single-target reaching task in monkey Q (*n* = 129 cells, average of 30 trials/conditions). In the left panel, the cells have been sorted, based on the amplitude of change in the Z-scored data. On the middle and right panel, cells are sorted in the same order as left panel. (B) Example of a direction-related cell, indicated by the arrowhead in (A), with a significantly higher increase of activity on the rewarded target’s presentation on the left. The cell’s activity is aligned on the rewarded target onset marked by the vertical dash line. Colored curves represent the average *Z* score activity ±SD, separately for rewarded target on the left (green, *p* = 0.03), central (red *p* = 0.92), and right (blue, *p* = 0.43). (C) Pie chart representing the proportion of cells that were not modulated, direction-related, or non-direction related (black, dark gray, and light gray, respectively). Significance evaluated with an FDR-corrected Wilcoxon signed rank test with *p* < 0.05. See also Figure S1.

We found that in monkey Q’s SMA, 34% (43/129) of cells significantly modulated their activity in response to the presentation of the rewarded target and the immediately ensuing movement (*p* < 0.05, Wilcoxon signed rank test, corrected for multiple comparisons), as shown in Figure 2A, where the average raw calcium traces of 129 cells (obtained across seven sessions) was aligned to target presentation. In 11/129 cells (9%), the activity was not direction-related, but 32/129 cells (25%) showed larger changes in activity in trials where the target was located at a specific position (Figures 2B and 2C). The magnitude of the responses did not differ between cells responding with an increase or decrease in firing (Mann-Whitney rank-sum test) and was similar regardless of the target position (Kruskal-Wallis test; data not shown).

In monkey U’s SMA, we found that 43% (6/14) cells had significant modulation of activity when the calcium traces were aligned to the appearance of the rewarded target, and in all cases (6/6) these changes in activity were related to the direction of the movement. These proportions were the same when the data were aligned to the movement onset (when the monkey removed the hand from the holding target).

In monkey U’s M1, when the calcium traces were aligned to the appearance of the rewarded target, 4/6 cells (67%) showed changes in activity, without directional relation. When the data were aligned to the movement onset, 100% (6/6) cells were modulated, and 50% of them showed direction-related activity (Figure S1).

These data indicate that the calcium imaging method allows identification of a subset of SMA and M1 neurons that are modulated in a direction-related manner when presented with a movement-related target in the reaching task.

### Cells in SMA and M1 show coactivation

**Figure 3:**
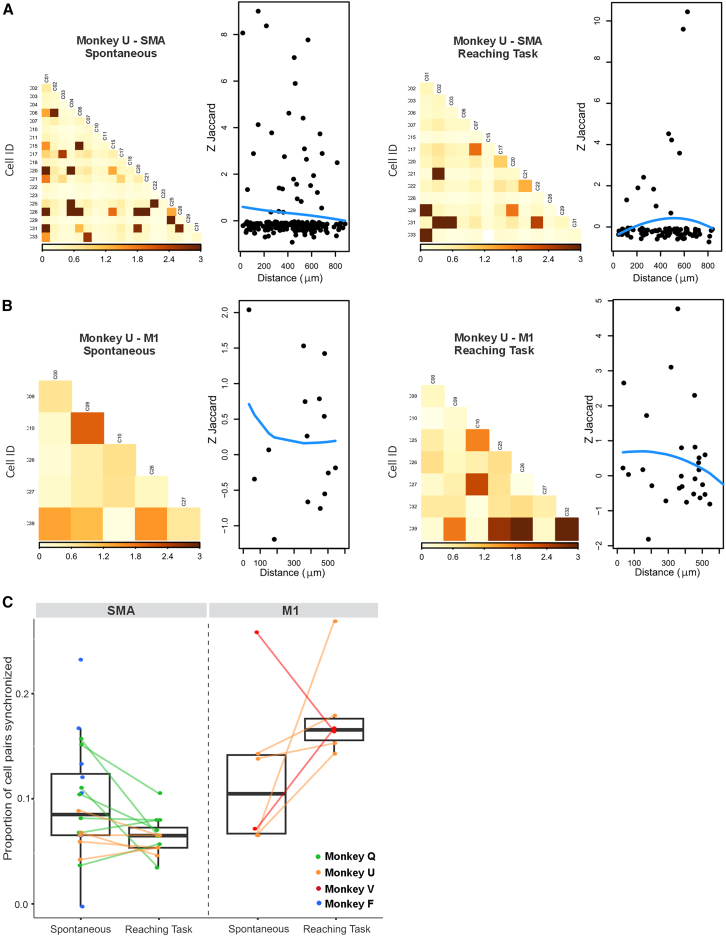
Coactivation between cell events in both SMA and M1 (A) Coactivation plots based on recordings made during the spontaneous and the task conditions in SMA in one example session from monkey U. The shading of squares represents the normalized Jaccard index (Z-Jaccard) for each cell pairing (darker means greater synchrony). On the right of each coactivation plot, we show the scatterplots in which each point represents the Z-Jaccard index for a pair of cells and the distance between their centroids. The blue line is the loess smoother (span = 1) used to visualize whether there is a relationship with distance. No clear patterns emerged. (B) Example session from monkey U in M1. Same legend as (A). (C) Boxplot summarizing the proportion of cell pairs synchronized during spontaneous and task conditions in SMA and M1. The horizontal bars represent the median value across all sessions and animals (monkeys Q, U, V, and F in green, orange, red, and blue, respectively). Each dot represents one session. Spontaneous and task values from the same session are connected by a line. Significance evaluated with a Wilcoxon signed rank test with *p* < 0.05. No significant differences were identified.

We studied the synchrony between pairs of cells using the Jaccard index, using denoised data. Example sessions are shown for monkey U in the SMA (Figure 3A) and M1 (Figure 3B) for spontaneous and task conditions. Pairs of neurons exhibited neuronal synchrony in both the SMA and M1 during the spontaneous and task conditions (Figures 3A and 3B). The cell pairs exhibiting synchrony were not related to the centroid distances in either SMA or M1 (correlation plot in Figures 3A and 3B). Figure 3C summarizes the proportion of synchronized pairs of cells (|Z-Jaccard| > 1.96) in the spontaneous and task condition recordings in SMA and M1 for all animals. The proportion of cells that were synchronized did not significantly differ between spontaneous and reaching tasks in either SMA or M1 (*p* = 0.07 and *p* = 0.22, respectively; Wilcoxon signed rank test); however, the number of sessions could be too low to produce significant differences. Cell-to-cell synchrony appears to be a reliable feature of SMA and M1 neurons, regardless of task and distance.

### Cells in SMA and M1 show sequential activation patterns

We then examined whether groups of neurons exhibited sequential activation patterns. A sequence was defined as a recurring series of calcium events involving at least two cells, occurring in a specific temporal order, and appearing at least four times within a 10-min recording. Sequences were only considered if the random occurrence probability was below 5% and if individual events were at least 0.05 s apart (to avoid detecting simultaneous bursts of events as “sequences”).

**Figure 4:**
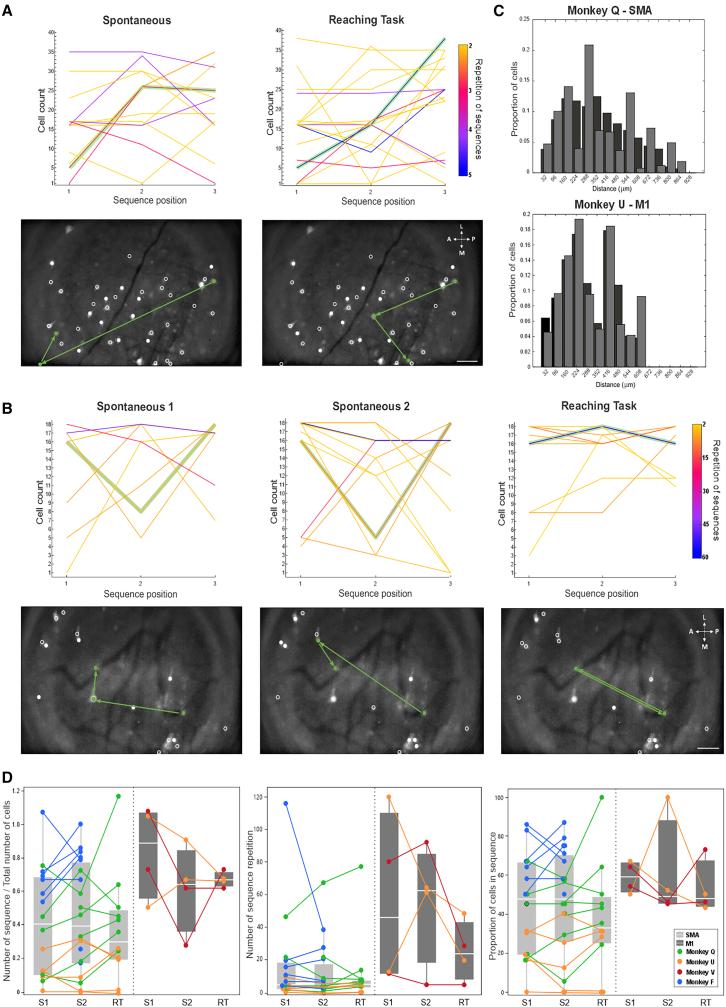
Repeating sequences of calcium events involving several neurons in the spontaneous condition and during the reaching task (A) Top row, depiction of sequences found across 40 cells in an example session in monkey Q’s SMA, during spontaneous and reaching task conditions. The *x* axis indicates the position of the cell within a sequence. The line thickness indicates the number of repetitions of the sequences. An example sequence starting with cell #5 is highlighted in green for the spontaneous and for the task condition. Below each graph we show the maximal GCaMP6f fluorescence intensity overlapped with the map of cells detected (white circles). The green circles illustrate the three cells involved in the sequence, and the green arrows indicate the temporal order. Although cell #5 initiates the sequence in both examples, it is followed by different cells in the spontaneous and reaching task. Scale bars: 100 μm. (B) Sequences found across 18 cells in a different session in monkey Q’s SMA, during two 10-min segments of the spontaneous conditions (“spontaneous 1,” “spontaneous 2”) and during the reaching task. Same conventions as described for Figure 4A. (C) Spatial distribution of cells involved in sequences. Black bars indicate the distribution of the centroids of all the cells recorded across all sessions in the spontaneous condition in monkey Q SMA (top) and monkey U M1 (bottom). Gray bars indicate the distribution of the centroids of the cells involved in sequences. Note overlap of the distributions, indicating that cells involved in sequences were not clustered but spread across the field of view. (D) Boxplots representing normalized number of sequences, number of repetition, and proportion of cell in sequence across monkeys in SMA (light gray) and M1 (dark gray). The number of sequences normalized by the total number of cells due to the different number of cells identified in each session. Each dot represents median values for a session; the horizontal bars represent the median value across all sessions and animals (monkeys Q, U, V, and F in green, orange, red, and blue, respectively). Data are binned in 10 min of recording in spontaneous (S1 and S2) and during the reaching task (RT). See also Figure S2.

In an example imaging session in monkey Q’s SMA, we detected 13 such sequences in spontaneous and 17 sequences during the task (Figure 4A, top panels). While some of the sequences detected involved the same cells (Figure 4A, bottom panels), the specific sequences differed (examples highlighted in green in Figures 4A and 4B). We also performed the same analysis for sessions in which 20 min of spontaneous recording was collected, allowing us to segment the spontaneous segment in two 10-min periods (“spontaneous 1,” “spontaneous 2”). An example from such a recording in monkey Q’s SMA is shown in Figure 4B. We detected 8, 13, and 9 unique sequences during “spontaneous 1,” “spontaneous 2,” and the reaching task, respectively. The detected sequences differed between all three states, suggesting that the differences between the analyzed segments were not (solely) driven by the behavioral state.

For all imaging sites, and for each recording segment, we calculated the number of sequences (normalized by the number of identified cells), the repetition rate of sequences, and the proportion of cells involved in sequences (summarized in Figure 4D). We found that these factors varied across conditions and across imaging sites, similar to observations made in monkey Q’s SMA.

Finally, we investigated whether cells participating in a sequence were spatially close. Plotting the distribution of distances between pairs of cells that were involved in sequences, as well as the overall distribution of distances between all detected cells showed that there is no preferred spatial relation between cells involved in sequences (Figures 4C and S2).

### Expression of GCaMP6f and lens localization

Postmortem histological examination confirmed that the lens and prism were placed in the arm regions of SMA and M1^8^^,^^13^^,^^14^^,^^15^ (Figure S3A). However, in monkey Q, the SMA lens was positioned at the anterior edge of this region, likely bordering with the pre-SMA area.^8^^,^^15^

The reconstruction of the location of the lens/prism showed that the probes were targeted to the deep cortical layers of the SMA/M1 (right panels in Figure S3A). GCaMP6f expression around the imaging sites was revealed by immunoperoxidase against green fluorescent protein (GFP) (Figure S3B) or by studying the endogenous fluorescence of the calcium sensor (Figure S3C). We observed robust GFP expression around the lens/prism for most sites, except in monkey U’s SMA, where the GFP expression was sparse. Importantly, many GFP-positive cells showed the morphology of pyramidal cells (i.e., excitatory projection neurons).^16^ We confirmed that GCaMP6f expression was specific for non-GABAergic neurons by performing a double immunofluorescence assay for GABA and GFP (Figure S3D), confirming that GCaMP6f-positive cells are likely to be projection neurons and not interneurons.

## Discussion

We examined the activity of projection neurons in deep layers of SMA and M1 of four rhesus macaques, when they were at rest (spontaneous condition) or performing a simple arm-reaching task, using calcium imaging techniques. As expected, based on the results of previous electrophysiologic studies, we found that a proportion of SMA and M1 neurons generated calcium transients in relation to arm reaches and that these responses were often directionally related. We also identified pairs of projection neurons in SMA and M1 with coincident activity and dynamically changing sequential activity patterns. Neither coincident nor sequence-related activities differed by spatial distances within the imaged field.

Our study complements the extensive literature on the electrophysiological activity of SMA and M1 neurons in macaques,^2^ and it builds upon previous studies that have used calcium imaging and miniscopes to examine motor cortex activity in rodents (e.g.,^17^^,^^18^). The calcium imaging method offers unique opportunities to increase our understanding of cortical activity in NHPs, allowing us to image many neurons in deep layers in M1 and SMA. As an important distinction to the previous electrophysiologic studies in rhesus monkeys, by combining a genetic strategy (use of the Thy1 promoter that targets preferentially projection neurons^12^) and placing the GRIN lenses in deep cortical layers, we selectively monitored cortico-fugal neurons.

We compared calcium activity from the spontaneous to the reaching task condition in individually identified neurons that were reliably tracked within a session. We found that neither the rate nor the variability of calcium events differed significantly between the two conditions, perhaps indicating that the information coded by these parameters is not indicative of the behavioral state of the animal. However, a previous microendoscopic calcium imaging study in the M1 of marmosets reported an increase in calcium event rates from rest to movement,^19^ a difference that could be due to the more complex tasks the marmosets were engaged in (reaching for food pellets or climbing ladders).

We found that calcium event rates were, in general, higher in M1 neurons than in SMA neurons, which differs from electrophysiology studies that report similar firing rates in both regions.^15^ The difference may be partially explained by the fact that the electrophysiologic measurements were not specific for corticofugal neurons. In addition, the two techniques detect different neuronal activity patterns: while electrophysiologic recordings identify both single spikes and bursts of firing, calcium imaging is more sensitive to bursts of activity.^20^ Our result may therefore reflect a difference in resting firing patterns between M1 and SMA rather than a different overall activity of neurons in these regions.

In our study, the calcium event rate was about 10-fold higher than previously reported in the premotor cortex of rhesus macaques.^9^ The rate of calcium events we detected was also different from that reported in the M1 of marmosets.^19^ These discrepancies are not only likely related to species differences (in the case of the marmosets) but also a direct result of the definition of “calcium events”: our calculation of the event rate is based on the detection of such events with a deconvolution method, unlike the study of Bollimunta et al.

In a subpopulation of SMA and M1 neurons, the calcium activity was related to arm reaches, and some neurons showed directional sensitivity, as previously observed with electrophysiological recordings.^2^^,^^8^^,^^15^

However, the proportions of SMA neurons (34% for monkey Q and 43% for monkey U) showing task-related responses were lower to previous electrophysiological reports where monkeys engaged in similar arm-reaching tasks (e.g., refs.^15^^,^^21^^,^^22^^,^^23^). This may have resulted from the placement of the lens in the most anterior portion of the SMA forelimb region, which has a patchier representation of the forelimb than the most caudal SMA.^8^^,^^15^ On the other hand, the proportion of neurons in M1 that showed task-related responses (100% when aligned on movement onset) and the proportion of those exhibiting directional sensitivity (50%) align with findings from previous electrophysiological studies,^15^^,^^23^^,^^24^^,^^25^ even though the total number of neurons imaged in this location was low.

Similar results were reported in another study using microscopic calcium imaging in the M1 of marmosets.^19^ Thus, even though the time resolution of calcium transients is orders of magnitude lower than electrophysiological recordings, the calcium imaging method can detect basic functional parameters of these cortical regions.

Microendoscopic calcium imaging can simultaneously monitor many cells in a relatively large area of cortex, allowing analysis of simultaneous and sequential activation of neurons. In SMA and M1, we found pairs of cells that were coactive, unrelated to their spatial proximity (within the confines of the evaluated imaging field). Our results in the macaque motor cortices differ from the findings of Parker et al., who reported coactivation in spatially close groups of striatal medium spiny neurons in rodents,^26^ likely due to different structural organization across brain regions.

In a related analysis, we found that the activity of neurons in M1 and SMA can form recurring sequential patterns, involving multiple cells. Similarly to the findings of the coactivation analysis, the distance between neurons did not predict their involvement in sequences. The sequences were not stable over time, and not specifically dependent on the behavioral context of the recordings, as variations of patterns were also seen when comparing different segments of recordings done in the spontaneous condition. We could not derive a true estimate of the variability of the statistically identified sequences because the low calcium event rates preclude an examination of these sequences in short time intervals. Given that the identified sequence interactions occurred over relatively long periods (up to 2 s), they are not likely to be the product of local neuronal interactions but may represent long-range multi-synaptic networks.

As far as we know, this is the first report of the use of calcium imaging to examine neuronal sequential activation patterns in rhesus macaques. Following similar goals, studies of birdsong development have identified sequential neuronal activation patterns with calcium imaging.^27^^,^^28^ In those studies, sequential patterns were linked to the learning of precise vocal patterns. In our case, we did not find a clear correlation between sequential patterns and behavior (spontaneous or reaching task), suggesting that the detected sequences could be related to functional processes that were not directly assessed in our experiments, such as motivational states, or cognitive processes. Consistent with this interpretation, previous work has shown that hippocampal neurons engage in precisely timed sequences even when animals are not actively performing a task (for example, in the delay period of a maze run task), suggesting that such sequences may encode future behavioral intentions.^29^ However, such links remain speculative at this time.

### Limitations of the study

The calcium imaging technique has some important limitations. The temporal resolution of the calcium transient imaging procedure is much lower than that of electrophysiological recordings, limiting the analysis of neuronal events that occur at faster time scales. One reported advantage of the calcium imaging technique using miniscopes is the ability to track the same cells across days,^30^ based on the spatial stability of fluorescence and surrounding tissue landmarks within the imaged field. However, in our study we were not able to routinely track neurons across multiple sessions. This issue was also reported in studies conducting calcium imaging in the M1 of rats.^31^ Several factors may impact the ability to longitudinally track calcium transients in the same cells. One factor could be that the expression of GCaMp6f is unstable, but this seems unlikely, as our postmortem studies showed robust calcium sensor expression even after survival times of up to 189 days following viral vector injections. Alternatively, changes in the field of view, due to movement of the brain tissue in relation to the GRIN lenses, may interfere with longitudinal recordings. It is conceivable that this may differ between animal species or even between brain regions within the same animal.

The sparse GCaMp6f expression in monkey U’s SMA may explain the low number of cells imaged in this animal. However, the GCaMP6f expression levels observed postmortem in monkeys F and V and in monkey U’s M1 were much higher, but the number of cells imaged *in vivo* were also found to be comparatively low. Overall, the total number of neurons imaged per session in our studies ranged from 23 to 129 (Table S2), which contrasts to a reported average of 90 cells in the rat M1.^31^ This difference may reflect the fact that the neuron density is lower in the primate M1 compared to that of the rodent M1.^32^^,^^33^

In summary, we used microendoscopic calcium imaging to study groups of neurons in the SMA and M1 in rhesus macaques, across behavioral states. Our study sets the stage for use of this method to examine calcium dynamics in groups of cells in the primate motor cortices in more complex and controlled behavioral tasks, to compare patterns of activity among cells in disease states, such as parkinsonism,^34^ and to study neuronal responses to acute or chronic treatments in these models. Finally, given the ongoing development of enhancer sequences that can be used in AAVs or other viral vectors,^35^ it may soon be possible to expand this technique to track the activity of other neuronal subtypes (e.g., subtypes of cortical inhibitory interneurons or specific cortical projection neurons).

## Resource availability

### Lead contact

Further information and requests for resources should be directed to and will be fulfilled by the lead contact, Anne-Caroline Martel (amarte4@emory.edu).

### Material availability

This study did not generate new materials.

### Data and code availability

• Data: All data generated in the study have been deposited at DANDI and/or Zenodo and are publicly available as of the date of publication. The corresponding DOIs are listed in the key resources table.
• Code: All original code used in the study have been deposited at Zenodo and are publicly available as of the date of publication. The corresponding DOIs are listed in the key resources table.
• Any additional information required to reanalyze the data reported in this paper is available from the lead contact upon request.

## Acknowledgments

We are grateful to Wendy Williamson Coyne for assistance during surgeries, to Susan Jenkins, DeErra Locklin, and Charlotte Armstrong for technical help, and to Natalia Magnusson for the design of the graphical abstract. Some of the surgeries described in this study were conducted in collaboration with the Emory Translational Neuroscience Core, which is supported by the Department of Neurosurgery, Emory University School of Medicine.

Funding: This research was funded by Aligning Science Across Parkinson’s (ASAP-020572) through the Michael J. Fox Foundation for Parkinson’s Research (MJFF) and by 10.13039/100000002NIH P51-OD011132 (Emory National Primate Research Center).

## Author contributions

Conceptualization, J.J.N., T.W., and A.G.; methodology, J.J.N., W.Y., J.D., T.W., and A.G.; software, B.R., A.D., and T.W.; investigation, A.C.M., D.P., and A.G.; formal analysis, A.C.M., D.P., A.D., B.R., and T.W.; writing—original draft, A.C.M., D.P., and A.G.; writing—review & editing, A.D., B.R., J.J.N., W.Y., and T.W.; visualization, A.C.M., D.P., A.D., B.R., T.W., and A.G.; supervision, T.W. and A.G., funding acquisition, T.W. and A.G.

## Declaration of interests

W.Y., J.D., and J.J.N. are paid employees of Inscopix, Inc. The remaining authors declare no competing interests.

## STAR★Methods

### Key resources table

**Table:** 

REAGENT or RESOURCE	SOURCE	IDENTIFIER
**Plasmids**		

pAAV TREGCaMP6f	Addgene	Cat # 97410
pAAV ThyStTA	Addgene	Cat # 97411

**Viral vectors**		

AAV5-TRE-GCAMP6f	Vigene Biosciences	Quote # 012022067
AAV5-ThyStTA	Vigene Biosciences	Quote # 012022067

**Antibodies**		

Chicken anti-GFP (immunofluorescence)	Millipore	Cat # 06-896; RRID: AB_310288
Rabbit anti-GFP (immunoperoxidase)	Thermo Fisher (Invitrogen)	Cat # A-11122; RRID: AB_221569
Mouse anti-MAP2	Millipore	Cat # MAB3418; RRID: AB_11212326
Rabbit anti-GABA	Sigma	Cat # A2052; RRID: AB_477652
Biotinylated goat anti-rabbit	Vector	Cat # BA1000; RRID: AB_2313606
Biotinylated goat anti-mouse	Vector	Cat # BA9200; RRID: AB_2336171
Donkey anti-chicken (Fluorescein)	Jackson Lab	Cat # 703-095-155; RRID: AB_2340356
Donkey anti-rabbit (Rhodamine red-X)	Jackson Lab	Cat # 711-295-152; RRID: AB_2340613

**Experimental models: Organism/strains**		

Rhesus macaques (*Macaca mulatta*)	Emory National Primate Research Center, Atlanta, GA, USA.	N/A

**Protocols**		

Surgery to inject viral vectors and implant GRIN lenses	This study	Protocols.io: https://doi.org/10.17504/protocols.io.e6nvw15w2lmk/v1
Miniscope calcium imaging data acquisition of cortical activity	This study	Protocols.io: https://doi.org/10.17504/protocols.io.14egn612pl5d/v1
Arm reaching to touchscreen behavioral task	This study	Protocols.io: https://doi.org/10.17504/protocols.io.5jyl8d41rg2w/v1
Immunoperoxidase staining	This study	Protocols.io: https://doi.org/10.17504/protocols.io.14egn2b6mg5d/v1

**Software and algorithms**		

IDPS	Inscopix	https://inscopix.com/software-analysis-miniscope-imaging/
MATLAB, v 2023b	MathWorks	RRID: SCR_001622 https://www.mathworks.com/
R v.4.4.1	R Core Team	RRID: SCR_001905 https://www.r-project.org/
CorelDraw, v2019, v2024	Corel	RRID: SCR_014235 https://www.coreldraw.com/
Sequence detection (MATLAB code)	This study	Zenodo: https://doi.org/10.5281/zenodo.14963234
Event based analysis (MATLAB code)	This study	Zenodo: https://doi.org/10.5281/zenodo.14963213
Jaccard analysis (MATLAB code)	This study	Zenodo: https://doi.org/10.5281/zenodo.14977106
MonkeyLogic code	This study	Zenodo: https://doi.org/10.5281/zenodo.15346239

**Deposited data**		

One-photon microendoscopic raw data, CNMFE extracted maps and time series	This study	DANDI archive: https://doi.org/10.48324/dandi.001174/0.250331.2218
General descriptors of calcium activity in M1 and SMA cells	This study	Zenodo: https://doi.org/10.5281/zenodo.14727695
Cell activity aligned to behavioral events	This study	Zenodo: https://doi.org/10.5281/zenodo.14976651
Cell coactivation (Jaccard) analysis outputs	This study	Zenodo: https://doi.org/10.5281/zenodo.15116617
Sequence analysis outputs	This study	Zenodo: https://doi.org/10.5281/zenodo.14976005
Histology images	This study	Zenodo: https://doi.org/10.5281/zenodo.15328390

### Experimental model and subject details

Four rhesus macaques (Macaca mulatta) were studied (monkeys F, Q, U, and V, 2 males, 2 females). The animals were 4–5 years old and weighed 5.5–6.8 kg at the beginning of the study. The monkeys were pair-housed in a temperature-controlled room with a 12-h light cycle, fed a standard primate diet twice daily with additional fruit and vegetable supplements, and had access to water *ad libitum*. All procedures were approved by the Institutional Animal Care and Use Committee (IACUC) of Emory University and were performed according to the Guide for the Care and Use of Laboratory Animals and the U.S. Public Health Service Policy on the Humane Care and Use of Laboratory Animals.

### Method details

#### Behavioral task

Two of the monkeys (Q and U) were first trained to accept head fixation with a thermoplastic molded helmet (CIVCO medical solutions)^36^ or a surgically implanted head post and then trained on an arm reaching task (NIMH, MonkeyLogic v2.2). The monkeys were facing a 17-inch display touchscreen (GVision) during task performance. Training began 2–4 weeks before lens implant surgery (see below) and continued until the animals reached criteria (see below).

Monkey Q was trained in a one-target task that required the animal to touch a circle (∼93 mm diameter) shown on the touchscreen display with its right hand to receive a juice reward. For each trial, the target would randomly appear on the right, left, or center of the touchscreen and remained on display for up to 3 s. If the monkey touched it during this period (successful trials), the target disappeared, and the animal received a drop of juice. A new trial started after a random inter-trial interval (2–3 s), following the release of the monkey’s hand from the touchscreen.

Monkey U was trained in a two-target task, in which the monkey had to first touch a center target (circle, ∼52 mm diameter). After the monkey held the center target for 1 s, targets on the left or right side of the screen (randomly chosen) would appear. The monkey had to release the center target within 1 s and touch the side target (successful trial), to receive a drop of juice. The monkey worked with its right hand for the imaging sessions evaluating the left SMA, or with his left hand for the imaging sessions focused on the right M1.

We considered monkeys trained in these tasks when they consistently completed at least 100 successful trials per 10-min session.

#### Surgery (AAV injections and lens implant)

Monkeys were subjected to a surgery to receive injection of virus solutions and implantation of GRIN lenses, following the surgical protocol listed in the key resources table and Bollimunta et al.^9^ The location of the lens implants was determined based on pre-surgery MRI scans and stereotaxic coordinates obtained from Paxinos et al.^37^ The surgery was performed under sterile conditions. Monkeys were sedated with ketamine (10 mg/kg), followed by isoflurane anesthesia (1%–3%) for the remainder of the procedure. They were placed in a stereotactic frame, and their vital signs were monitored throughout the surgical procedure.

We performed two craniotomies, each producing skull defects measuring approximately 10 mm in diameter, over the arm regions of the left SMA and the right M1. Following the craniotomies, we made a small incision in the dura mater for virus injections and GRIN lens implants at each cortical site.

We used a virus solution consisting of a 1:1 mixture of AAV5-TRE-GCAMP6f (9.05 × 10e13 gc/ml) and AAV5-ThyStTA (5.13 × 10e13 gc/ml). The Tet-Off system strategy was used because the GCaMP6f expression is amplified due to the tetracycline (tTA) promoter activation of the tetracycline response element (TRE3).^9^^,^^19^ In addition, if needed, GCaMP6f expression could be temporarily suppressed with systemic administration of doxycycline.^9^ Note, however, that doxycycline was not used in these studies. A Hamilton syringe with a 25-ga needle was filled with the mixture and then lowered into the brain using an injection pump mounted to the stereotactic frame (Stoelting Quintessential stereotaxic injector). We started injections of the virus solution after a 3-min wait time.

We injected 3–6.4 and 5–7.8 μL (at 0.1–0.8 μL/min) of virus solution in M1 and SMA, respectively. Information about the depths and volumes of injections in each site is provided in supplementary material (Table S3). The needle remained at each injection site for at least 3 min after the final injection.

After the virus injection, an epoxy-filled 18-ga needle was lowered into the brain to create a path for the subsequent insertion of a GRIN prism for M1 (1 mm diameter, 9 mm, or 12 mm length), or a flat lens for SMA (1 mm diameter, 12 mm length), pre-attached to a baseplate to dock a miniscope (ProView Integrated Probe or ProView Integrated Prism Probe; Inscopix). In both brain regions we aimed to implant the lenses at a vertical or nearly vertical orientation (for ease and stability of later microscope docking) and image perpendicular to the plane of the cortical surface. For M1, which sits on the gyral surface of the cortex, a prism is required to attain the intended imaging field of view. For SMA, which sits in the cingulate sulcus along the medial wall of the brain, no prism is necessary and therefore we used a flat lens. The baseplate with the integrated lenses were lowered into the brain to target the center of the dorsal-ventral extent of the virus injections. Following the lens implantation, a cranial chamber was positioned around each baseplate-integrated lens, affixed to the skull, and capped for protection. The exposed brain tissue around the lens was covered with gel foam and a slotted aluminum disk. The implant was secured to the disk and skull within the chamber with Metabond dental cement (Parkell, Edgewood, NY) or Gradia Direct Flo light-cured composite (GC Corp., Tokyo Japan). Finally, the exposed skull was covered with bone screws and dental acrylic. We then secured a head holder in the dental acrylic cap.

#### Calcium imaging data acquisition

Nineteen to 48 days post-surgery, we started imaging calcium transients in SMA or M1 neurons using a head-mounted miniscope (nVista3.0, Inscopix, CA, USA). We did not perform simultaneous recordings in both regions. The miniscope was docked to the integrated baseplate of the implanted lenses. Imaging data for each site were acquired at least twice weekly. During these sessions, the monkeys were sitting awake on a primate chair (confirmed by continuous monitoring via a live video stream). Head movement was restricted by a thermoplastic helmet or by a head post. In each session, we performed 10–20 min of imaging in the “spontaneous” condition during which the monkeys performed only occasional spontaneous movements of arms, trunk, and legs, followed by imaging while the animals worked on the “reaching task” for as long as it took to complete 100 successful trials (median 12 min, inter-quartile range (IQR) 3.5 min). For some of these recordings, TTL pulses from the behavioral task, indicating the occurrence of behavioral events, were recorded on additional channels. The miniscope remained mounted until the end of the session. Imaging and recording parameters were controlled using the Inscopix Data Acquisition Software (IDAS, Inscopix; frame rate, 10 Hz; LED power, 0.6–0.8 mW/mm^2^; sensor gain, 7–8X; and electronic focus, 400–900).

#### Histology

Monkeys were euthanized at the conclusion of the experiments with an overdose of pentobarbital sodium (100 mg/kg, iv). The post-surgery survival times were 189 days for monkey Q, 121 days for monkey U, 118 days for monkey V and 78 days for monkey F. They were then transcardially perfused with oxygenated Ringer’s solution, followed by 2 L of fixative [4% paraformaldehyde, 0.1% glutaraldehyde in phosphate buffer (0.1 M, pH 7.2)]. The fixed brains were then removed from the skull and blocked. The blocks were later cut into 60 μm slices using a vibratome and immunostained for the neuronal marker microtubule-associated protein 2 (MAP2), and for green fluorescent protein (GFP) to examine lens placement and GCaMP6f expression in neuronal cell bodies of projection neurons, respectively. In addition, to confirm the identity of the GCaMP6f expressing neurons, we performed double fluorescent immunolabeling for GABA (as a marker of interneurons). See “key resources table” for antibody details.

### Quantification and statistical analysis

#### Cell identification and trace extraction

Raw calcium imaging recordings, comprised of spontaneous and reaching task conditions, were imported into the IDPS (Inscopix Data Processing Software) for cell identification and trace extraction. The recordings were preprocessed by removing excess pixels, correcting defective pixels using a 3 × 3 median filter, and performing 4-fold spatial down sampling of each image frame. We then applied a spatial band-pass filter (low and high cut-off points of 0.005 pixel^−1^ and 0.5 pixel^−1^, respectively) and performed rigid translation motion correction with a mean projection image as the global reference frame (to which other frames were aligned). Rigid motion correction is typically sufficient for one-photon miniscope imaging, given the relatively uniform motion of the brain, as shown in a previous paper using calcium imaging in macaques that were not head fixed.^9^ In addition, to further assess large z-motion, we looked for stable spatial footprints of calcium signals and lack of obvious inconsistencies in anatomical landmarks (i.e., abrupt, noise-like jumps in calcium traces).

Following that, we used the constrained nonnegative matrix factorization for microendoscopic data (CNMFe) algorithm^38^ to extract the calcium transients of individual cells. From the extracted calcium traces, we identified and accepted cells based on imaging metrics such as the cell’s spatial footprint, size, and signal-to-noise ratio. Profiles that did not have a somatic appearance (e.g., blood vessels or dendritic segments) or calcium dynamics were rejected from further analysis. On accepted cells, we deconvolved the traces using the “Online Active Set methods for Spike Inference” (OASIS) method^38^^,^^39^ to obtain the calcium events per cell. For each recording, we calculated the distance between the cells based on their spatial centroids (X Y coordinates). All data were further analyzed in MATLAB R2023b and R (v 4.4.1). We only report results for the first session in which cells were identified.

#### Statistical comparisons of calcium activity rates and amplitudes

For each identified cell, we calculated calcium event rates, the coefficient of variation of the inter-event intervals (IEI CV), and the mean amplitude of events during spontaneous and/or reaching task recording conditions. Statistical comparisons, contrasting the calcium event rates in the spontaneous and reaching task conditions in the SMA or M1, were performed using the Wilcoxon signed rank test. We were also able to compare data recorded in M1 and SMA (in monkey U only) in either the spontaneous or task conditions. These comparisons were performed using Wilcoxon rank sum tests. Additionally, we corrected for multiple comparisons using Benjamini-Hochberg false discovery rate (FDR).^40^ Statistical significance was set at *p* < 0.05.

#### Alignment to behavioral events

We used TTL pulses generated during the behavioral task to align raw calcium traces to behavioral events, using a custom-written MATLAB algorithm. We analyzed data from seven recording sessions in monkey Q’s SMA, as well as one session in SMA and one in M1 from monkey U.

Since each monkey performed a slightly different task (monkey Q performed a one-target task, while monkey U was trained in a two-target task), we first aligned the calcium traces to the event common to both tasks: the appearance of the rewarded target that prompted a reaching movement for a juice reward. We considered this event a suitable proxy for movement onset, as the monkeys initiated an arm movement to touch the target upon its appearance on the screen. For monkey Q, this target was the first and only one in each trial, whereas for monkey U, the rewarded target was the second one, following a required hold period at the center target (see behavioral task). For each successful trial, the 3-s epoch of raw data starting 1 s before target appearance was Z-scored to the first second of the 2 s inter trial period.

Additionally, for monkey U we aligned the calcium traces to the moment when the monkey removed its hand from the holding target and began reaching for the rewarded target. This event may have more precisely reflected movement onset. In this case, we also Z-scored a 3-s epoch of raw data to the inter trial period.

For both analysis (alignment to the appearance of the rewarded target and alignment to movement onset), the mean and standard deviation of Z-scores were then calculated for each cell and trial conditions (target on the right, center, or left for monkey Q, and target on the right or left for monkey U). Results from example sessions are shown in Figures 2 and S1. Cellular activity was considered as being modulated by the task events if the mean calcium activity during the event window (0–1 s after the reward target appeared for both animals, or 0–1 s after the hand left the center target for monkey U) differed significantly from the mean activity during the second preceding the target presentation or movement onset. We used Wilcoxon signed rank tests with multiple comparisons corrected at the level of each chamber using FDR. In the SMA of monkey Q, 129 cells were tested across 3 conditions (387 tests); for monkey U, 14 cells were tested in 2 conditions (28 tests) in SMA; and 6 cells were tested in 2 conditions (12 tests) in M1. Cellular activity was considered related to movement direction if only one condition was significant at FDR-corrected *p*-value <0.05, while non-directionally related neurons were those with a significant response to two or more conditions. The absolute value of the amplitude of change in the Z-scored signal between the 1-s preceding the target presentation and the 1-s following the target presentation was also extracted. The magnitude of change in cells showing an increase in activity, and those showing a decrease were compared using a Mann-Whitney rank-sum test and comparisons across condition (right, center, or left target) were made with a Kruskal-Wallis test. In all cases, significance was assumed if *p* < 0.05.

#### Cell coactivation (Jaccard index method)

We examined the synchrony between calcium events using the Jaccard index (implemented using custom R code). Using the deconvolved calcium event data exported from IDPS, we analyzed all sessions from each site (SMA and/or M1). Our calculation of the Jaccard Index follows Parker et al.^26^. First, we binned the deconvolved calcium events data into 0.2 s intervals, resulting in the number of bins ranging from 1508 to 6547 (median = 6012) and 2371 to 6041 (median = 3700) for the spontaneous and reaching task data, respectively. Then, we forward-smoothed that data by setting a bin equal to one if a calcium event occurred at any time during the current bin or four bins ahead (a total of 1 s). Specifically, let $y_{c,t}$ be the 0.2 s resolution event data, where c=1,…,C denotes the cell index and t=1,…,T denotes the time point. Then the forward-smoothed data is defined as $y_{c,t}^{*}=1$ if $y_{c,t}=1$, $y_{c,t+1}=1$, $y_{c,t+2}=1,y_{c,t+3}=1\text{,}$ and/or $y_{c,t+4}=1$. We then calculated the Jaccard index between all cell pairs as the intersection of the number of forward-smoothed events divided by the union. Let *c* and *c*’ denote two different cells. Then

$${Jaccard}_{c,c^{\prime }}=\frac{\sum_{t}y_{c,t}^{*}y_{c^{\prime },t}^{*}}{\sum_{t}y_{c,t}^{*}+\sum_{t}y_{c^{\prime },t}^{*}-\sum_{t}y_{c,t}^{*}y_{c^{\prime },t}^{*}}\text{.}$$

Then for each cell pairing, we generated the null distribution of Jaccard indices by randomly circularly shifting one of the cell’s time series and recalculating the Jaccard Index. Specifically, let *k* be a randomly selected index from 1,…,T. Then define the circularly shifted data: $y_{c^{\prime },t}^{\left(k\right)}=\left[y_{\;c^{\prime },k}^{*},\ldots ,y_{c^{\prime },T}^{*}{,y}_{c^{\prime },1}^{*},\ldots ,y_{c^{\prime },k-1}^{*}\right]\text{.}$ Then we calculated ${Jaccard}_{c,c^{\prime }}^{\left(k\right)}=\frac{\sum_{t}y_{c,t}^{*}y_{c^{\prime },t}^{\left(k\right)}}{\sum_{t}y_{c,t}^{*}+\sum y_{c^{\prime },t}^{\left(k\right)}-\sum_{t}y_{c,t}^{*}y_{c^{\prime },t}^{\left(k\right)}}\text{.}$

The circular shift preserves the autocorrelation structure of the forward-smoothed event data. This was repeated 1,000 times for each cell pair. Next, we calculated a normalized version of the Jaccard index, which we refer to as the Z-Jaccard:

$${Z\_Jaccard}_{c,c^{\prime }}=\frac{\left\{Jaccard_{c,c^{\prime }}-mean\left({Jaccard}_{c,c^{\prime }}^{\left(k\right)}\right)\right\}}{sd\left({Jaccard}_{c,c^{\prime }}^{\left(k\right)}\right)}\text{.}$$

Large positive values of Z-Jaccard indicate cells pairs whose events co-occur substantially more than random circular shifts of the data. Large negative values indicate cell pairs whose events co-occur less than expected by chance. We defined the proportion of cell pairs that were synchronized as the fraction of cell pairs in which the absolute value of the Z-Jaccard was greater than 1.96. For the sessions in which we had both spontaneous and reaching task condition, we compared whether there was a change in the averaged proportion of cell pairs synchronized using a Wilcoxon signed rank test.

We used the centroid information to examine whether there was a relationship between the Z-Jaccard and the distance between cells. A loess smoother (span = 1) was used to visualize the relationship between Z-Jaccard and distance.

#### Determination of precisely timed sequences of calcium events

Sequences of calcium events were detected using a custom-written MATLAB algorithm. Sequences of events were defined as the occurrence of two or more calcium spike events (based on the deconvolved data) in a defined temporal order (e.g., calcium event in neuron A - > calcium event in neuron B - > calcium event in neuron C - > among two or more cells, with each step of the sequence occurring within a specific time limit (2 s). We evaluated the occurrence rates of such sequences during 10 min segments of recordings during the spontaneous and arm-reaching task conditions. For this, the algorithm first tabulated the occurrence rates of all possible combinations of events within a given record. To test whether a specific combination of events occurred more frequently than would be expected by random alignment of the events, we generate 1,000 shuffled arrangements of the data, by shifting one of the data streams against the other, by random periods of time (using the MATLAB “circshift” routine). This procedure (largely) retains the temporal order of events within the data streams but disrupts the potential temporal coupling between them. Sequences that were detected in the original data were accepted as being significant if they occurred more commonly than seen in 95% of the shuffled data (thus, were outside of the 95th percentile of the shuffled data), if they were found at least 4 times during the 10 min observation period, and if individual occurrences were separated by at least 0.05 s (to avoid detecting simultaneous bursts of events as sequences). We then evaluated the number of “significant” sequences, the number of steps forming a sequence, the number of sequence repetitions, and the spatial distance of neurons participating in the sequence members (using the centroid values, see above).
