# Supplementary material for: Microendoscopic calcium imaging in motor cortices of macaques during rest and movement
Source: iScience. 2025 May 27;28(6):112767. doi: 10.1016/j.isci.2025.112767 (PMC12192352; doi:10.1016/j.isci.2025.112767)
Supplement: Document S1. Figures S1–S3 and Tables S1–S3 [file mmc1.pdf]

iScience, Volume *28*

Supplemental information

Microendoscopic calcium imaging

in motor cortices of macaques

during rest and movement

Anne-Caroline Martel, Damien Pittard, Annaelle Devergnas, Benjamin Risk, Jonathan J. Nassi, Waylin Yu, Joshua D. Downer, Thomas Wichmann, and Adriana Galvan

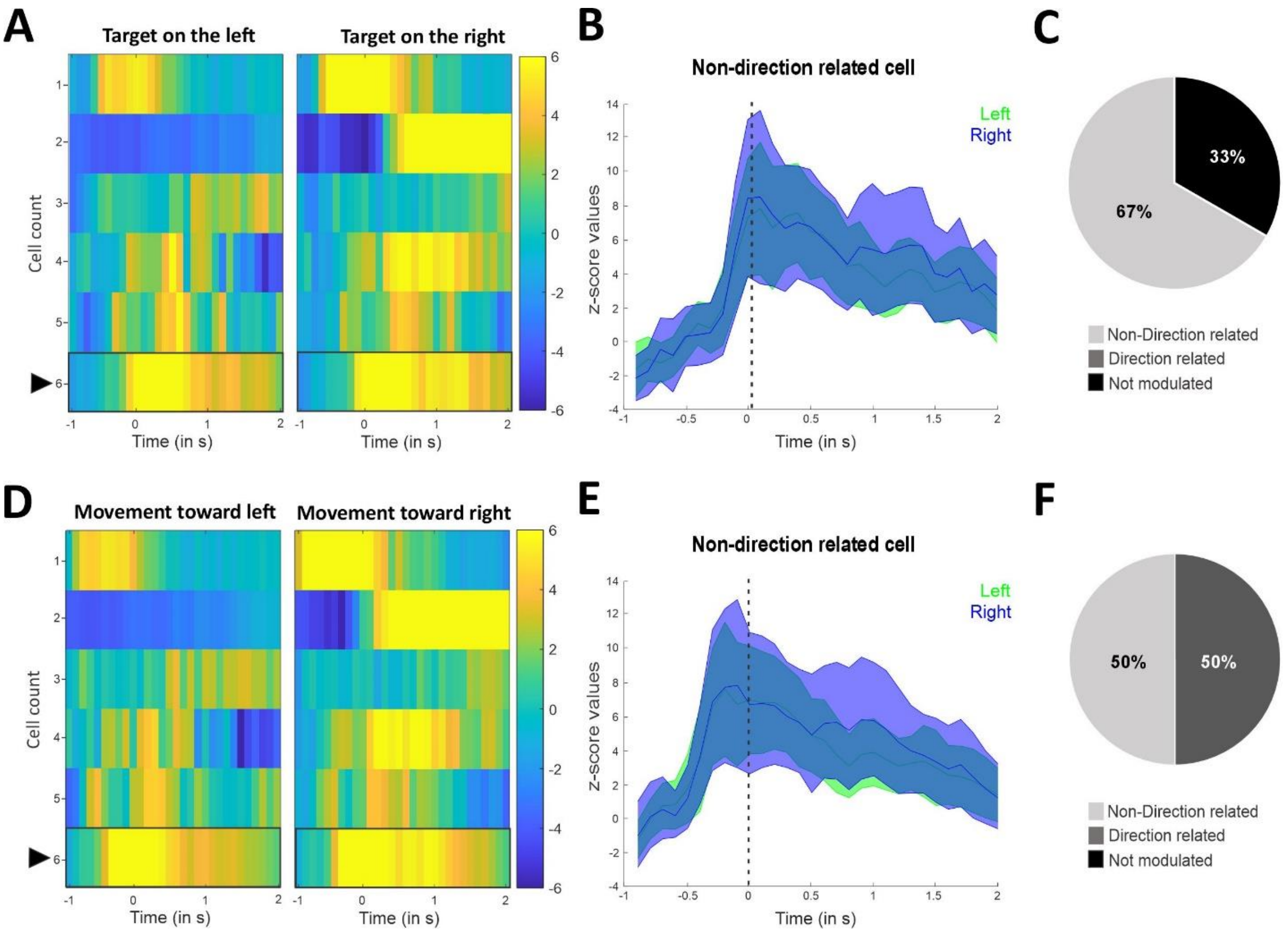

Figure S1: Changes in calcium activity in relation to rewarded target presentation and movement onset during an arm reaching task (related to Figure 2). A: Heat maps of the Z-scored raw calcium traces of each cell in an example session in M1, aligned on the rewarded target onset on the right or left during the two- target reaching task in monkey U (n=6 cells, 50 trials/condition). Each line indicates the same cell in both panels. **B:** Example of non-direction related cell, indicated by the arrow in A, with a significant increase toward both right and left rewarded targets ($p<0.01$ target left, $p<0.01$ target right, FDR-corrected Wilcoxon signed rank with $p<0.05$). The cell's activity is aligned on the rewarded target onset marked by the vertical dash line. Colored curves represent the average z-score activity ± SD separately for left (green), and right (blue) targets. **C:** Pie chart representing the proportion of cells that are not modulated (black), direction related (dark grey, none in this case) and non-direction related (light grey). **D:** Heat maps of the Z-score raw calcium traces of each M1 cell in the same session shown in A-C, but aligned on the movement onset toward the right or left cue in monkey U. The cells are sorted in the same order as in panel A. **E:** Example of the same non-direction related cell presented in panel B (indicated by an arrow in D), aligned on movement onset, showing a significant increase in activity toward both right and left ($p<0.05$ target right, $p<0.05$ target left, FDR corrected Wilcoxon signed rank with $p<0.05$). **F:** Pie chart representing the proportion of cells that are not modulated (black, none in this case), direction related (dark grey) and non-direction related (light grey).

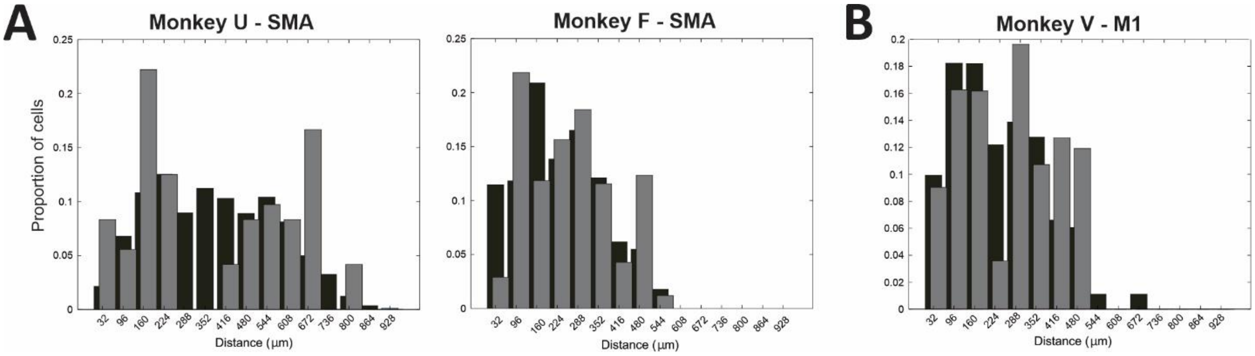

Figure S2: Spatial distribution of cells involved in sequences and sequence parameters (related to Figure 4). Black bars indicate the distribution of the centroids of all cells recorded across all sessions in the spontaneous condition for monkey U and F in SMA **(A)**, monkey V in M1 **(B)**. Grey bars indicate the distribution of the centroids of the cells involved in sequences. Note almost complete overlap of the distributions, indicating that cells involved in sequences were not clustered, but spread across the field of view.

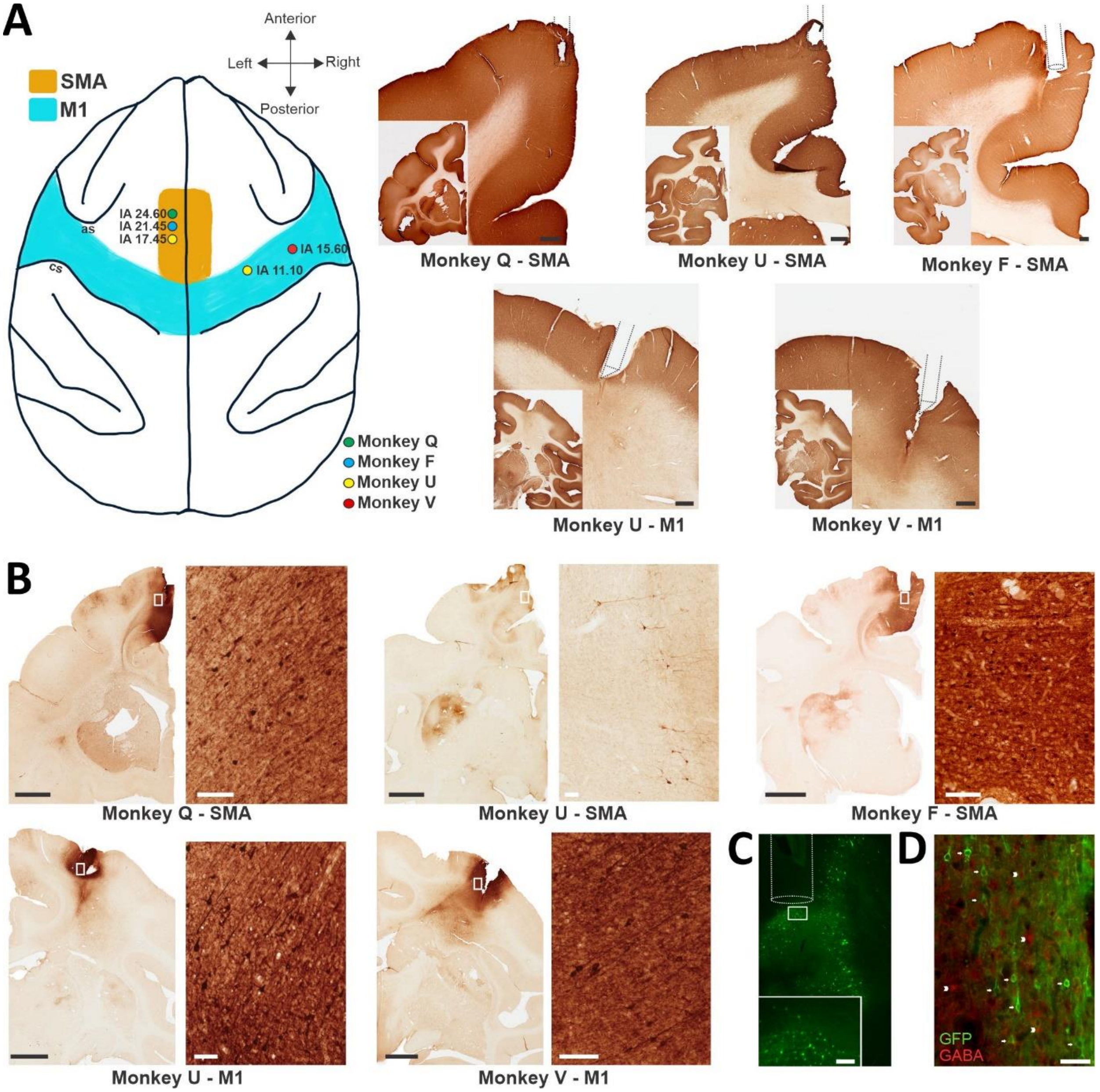

Figure S3: GCaMP6f expression and lens location in SMA and M1 (related to all figures). A: This diagram indicates the location of GRIN lenses, based on post-mortem verification in SMA and M1 for monkeys Q (green dot), F (blue dot), U (yellow dots) and V (red dot) with the corresponding interaural coordinates (based on the atlas by Paxinos et al 2000). Abbreviations: as, arcuate sulcus; cs, central sulcus. Right: Reconstructions of the lens placement in SMA (top row) and prism placement in M1 (bottom row) in MAP-2 stained brain sections. Black scale bars, 1 mm. **B:** GFP immunoperoxidase (to reveal GCaMP6f expression) in SMA and and M1. The white rectangles are shown at higher magnification on the right. Black scale bars, 4 mm; white scale bars, 100 μm. **C:** Endogenous green fluorescence of GCaMP6f in a section adjacent to those shown in A and B for monkey Q. Reconstruction of lens placement (white line), lens diameter is 1 mm. The white rectangle is shown at higher higher magnification on the right. White scale bar, 100 μm. **D:** Double immunofluorescence for GFP and GABA in the M1 of monkey U. Green indicates GFP (GCaMp6f)-positive cells, some indicated by arrows). Red indicates GABA-positive cells (some indicated by arrowheads). No overlap of the signals was observed, suggesting that GCaMp6f-expressing cells are not GABAergic interneurons. White scale bar, 50 μm.

Preprocess			Spatial Filter		Motion Correction		
**Spatial Downsampling**	**Fix defective pixels (Yes/No)**	**Trim early frames (Yes/No)**	**Low**	**High**	**Global ref. frame**	**Use ROI (Yes/No)**	**Preserve input dimension (Yes/No)**
4	Yes	Yes	0.005	0.5	Mean Image	Yes	Yes
**CNMFe Cell Identification**					**OASIS Deconvolution**		
**Avg. Cell Diameter (pixels)**	**Min. Pixel correlation**	**Min. Peak to Noise ratio**	**Guaussian filter width (pixels)**	**Merging threshold**	**Spike SNR Threshold**		
7-20	0.75 - 0.80	8-10	8-20	0.3	2.5		

Table S1: Parameters for cell identification and deconvolution (related to Figure 1A-C)

Animal ID	Structure	Imaging period (days)*	Number of sessions	Cells identified per session (median (range))	Total cells identified across sessions	Cells identified in more than one session
Q	SMA	147	7	18 (12- 40)	129	23
F	SMA	36	9	12 (1 - 16)	61	17
U	SMA	50	5	16 (10 - 25)	63	15
U	M1	35	4	8 (6 - 21)	37	6
V	M1	21	2	12.5 (11 - 14)	23	1
*Indicates days during which GCaMp6F positive cells were imaged						

Table S2: Number of sessions and cells identified per imaging site (related to Figure 1D-F)

Site	No. of injection tracks	Number of deposits	Deposit dorso-ventral location (mm)	Volume delivered per deposit (ml)
Monkey Q SMA	1	3	7, 6.5, 5.5	2.3, 1.8, 1.4
Monkey Q M1	1	2	4, 3	2, 1.3
Monkey U SMA	1	3	4.9, 4.4, 3.4	2, 1.5, 1.5
Monkey U M1	1	2	3.7, 2.7	1.5, 1.5
Monky V SMA	1	3	5.8, 5.3, 4.3	2, 1.5, 1.5
Monkey V M1	1	2	5.3, 4.3	1.5, 1.5
Monkey F SMA	1	4	5.5, 5, 4.5, 4	1.6, 1.6, 3, 1.6
Monky F M1	1	4	6.7, 6.2, 5.2, 4.7	1.6, 1.6, 1.6, 1.6

Table S3: Details of AAV injections (related to STAR methods)
